# The concise measurement of clinical communication skills: Validation of a short scale

**DOI:** 10.3389/fpsyt.2022.977324

**Published:** 2022-10-12

**Authors:** Ulrike Maaß, Franziska Kühne, Peter Eric Heinze, Destina Sevde Ay-Bryson, Florian Weck

**Affiliations:** ^1^Department of Clinical Psychology and Psychotherapy, University of Potsdam, Potsdam, Germany; ^2^Department of Rehabilitation Sciences, Humboldt-Universität zu Berlin, Berlin, Germany

**Keywords:** standardized patient, treatment integrity, measurement, therapist competence, role-play, psychotherapy process

## Abstract

**Objective:**

There is a lack of brief rating scales for the reliable assessment of psychotherapeutic skills, which do not require intensive rater training and/or a high level of expertise. Thus, the objective is to validate a 14-item version of the Clinical Communication Skills Scale (CCSS-S).

**Methods:**

Using a sample of *N* = 690 video-based ratings of role-plays with simulated patients, we calculated a confirmatory factor analysis and an exploratory structural equation modeling (ESEM), assessed convergent validities, determined inter-rater reliabilities and compared these with those who were either psychology students, advanced psychotherapy trainees, or experts.

**Results:**

Correlations with other competence rating scales were high (*r*s > 0.86–0.89). The intraclass correlations ranged between moderate and good [ICC_(2,2)_ = 0.65–0.80], with student raters yielding the lowest scores. The one-factor model only marginally replicated the data, but the internal consistencies were excellent (α = 0.91–95). The ESEM yielded a two-factor solution (*Collaboration* and *Structuring and Exploration Skills*).

**Conclusion:**

The CCSS-S is a brief and valid rating scale that reliably assesses basic communication skills, which is particularly useful for psychotherapy training using standardized role-plays. To ensure good inter-rater reliabilities, it is still advisable to employ raters with at least some clinical experience. Future studies should further investigate the one- or two-factor structure of the instrument.

## Introduction

One of the main goals of psychotherapy training is to improve trainees’ skills (1, 2). In order to identify these skills and monitor their changes as recommended by several authors (3, 4), valid and reliable measurement methods are needed (5, 6). Such measurements should be suitable for a number of different training contexts, for example, for the use in role-plays with simulated patients—a training approach that is becoming increasingly important in psychotherapy training (7–11).

Role-plays are particularly appropriate for assessing “therapist competency” in the narrower sense, that is, the demonstration of therapeutic skills in controlled conditions. By contrast, the assessment of competences in real therapy sessions and of treatment delivery refers to “therapy quality” (11–14). Ottman et al. note that “the need for reliable, standardized methods to assess therapist competency prior to treating clients remains a significant gap in the literature” (p. 10). Although role-plays offer a number of benefits, such as fair competence tests and targeted training for difficult situations (8), it is not easy to evaluate those skills that only come into play during the course of therapy or against the background of a specific treatment strategy (e.g., case conceptualization, repairing relationship ruptures etc.). The measurement of competencies in role-plays is therefore either limited to very specific skills that are necessary for the particular simulated scenario (e.g., performing an exposure) or focuses on general skills that are observable across situations. These common skills are often referred to as common therapy factors and include such skills as interpersonal skills or communication (15). Consequently, most competence measures include at least one item relating to such common factors (11, 16). There is evidence emphasizing the relevance of communication skills in particular for the improvement of client outcomes in therapy (17–19). However, so far, there is a lack of reliable instruments assessing communication skills that can be applied to different role-play scenarios across situations. For example, Ottman et al. (11) found only eight studies (out of 43) that measured competencies in standardized role-plays, whereas most instruments were applied to the assessment of real therapy sessions.

For cognitive behavioral therapies (CBT), two of the most prominent competence rating scales are the Cognitive Therapy Scale (CTS) (20) and its revised version (CTS-R) (21). The CTS-R consists of 12 items, which use a comprehensive 7-point rating scale. While some items might be appropriate for assessment in role plays, such as “interpersonal effectiveness,” other items relate to overarching skills or very specific ones that are not observable in all situations, such as “agenda setting,” “homework setting,” or “conceptual integration.” In addition, the CTS and CTS-R have been criticized for having a number of limitations, for example, unclear definitions of the behavioral basis underlying each item, low content validity, item overlap, and concept overlap within items (6, 13).

Another newly developed rating instrument is the Assessment of Core CBT Skills Scale (ACCS) (22), which comprises 22 items with a 4-point anchored rating scale. The authors’ intention was to address some of the criticisms of the CTS-R, for example, by developing clearer behavioral anchors to reduce the evaluators’ room for interpretation. However, in terms of usability for competence assessment in role-plays, the measurement also includes too many situation-specific items (e.g., homework, assessing change, CBT interventions).

Both the CTS/CTS-R and ACCS have also been criticized for their time-consuming application and the costly training of raters that is needed to achieve high rating reliabilities (16, 23). However, high levels of inter-rater reliability are not always accomplished across studies (5, 11). One reason might be the varying degree of rater expertise. In line with that, Wu et al. (24) suggested that expert ratings should serve as a standard for adherence ratings. This might also be true for competence ratings. For example, Weck et al. (25) found that, while novice raters achieved satisfactory ICCs without significant differences to the ratings of experts, the concordance between expert and novice raters was only moderate. In addition, Kühne et al. (26) reported that raters with both more clinical experience and experience in using the corresponding rating scale achieved higher ICCs.

Thus, while the commonly used competence rating scales (e.g., CTS/CTS-R, ACCSS) are well validated, they also display some general limitations (i.e., heterogeneity in ICCs, high training and completion effort, high expertise level required) and specific restrictions (i.e., focus on specific CBT techniques rather than on common factors) for the application across different situations (e.g., in role-plays).

For these reasons, Kühne et al. (23) developed a competence rating scale, the Clinical Communication Skills Scale (CCSS), which is easy and quick to complete and covers basic counseling techniques. The CCSS examines basic psychotherapeutic communication skills such as, asking open-ended questions, expressing empathy, or exploring cognitions, emotions, and behaviors. It is an observation-based assessment of general and cognitive-behavioral skills in both real and simulated patient situations, and includes 37 items and a 4-point rating scale. It thereby focuses on common factors rather than specific CBT techniques. In contrast to the CTS/CTS-R and ACCS, the CCSS does not deploy a comprehensive rating scale with behavioral anchors. Instead, the items are short and behavior-based. Examples of items that demonstrate the behavior orientation are “gives the patient time to talk and to ask questions” or “summarizes interim results.”

In a sample of *N* = 209 lay-persons and psychology students, the CCSS revealed a unidimensional one-factor structure with an excellent internal consistency (α = 0.94). The instrument achieved moderate to high convergent validity with established rating instruments (e.g., communication item of the CTS: *r* = 0.59, empathy: *r* = 0.68) and a good differentiation between high and low levels of competence of the therapists being rated (23).

Thus, the CCSS is a promising instrument for application in training with standardized role-plays in particular. It assesses clinical communication skills which is seen not only as an important basic skill that therapists have to acquire in their training (1), but also as a common factor of most competence measurements (11) and a predictor of therapy success (19). However, the original validation of the CCSS has not yet provided inter-rater reliabilities. Furthermore, the high internal consistency justifies the reduction of the item number, which will in turn decrease the time raters need to complete the scale. Especially in contexts like research, supervision and training, shorter scales are often needed due to time restrictions. Generally, short scales are advantageous, because they ensure the representativeness of the construct of interest without content repetition (27) and do not tend to overestimate the internal consistency (28).

Consequently, the objective of this present study is to validate a short version of the CCSS (23) in the context of standardized role-plays. Furthermore, as an extension to the original study, we will calculate inter-rater reliabilities (i.e., intraclass correlations, ICCs) based on video recordings of therapists behavior in simulated therapy session segments (i.e., standardized role-plays). Finally, we evaluate the impact of rater expertise by comparing the ICCs of trained psychology students, advanced psychotherapy trainees, and licensed psychotherapists with each other.

### Research questions and hypotheses

Our research questions were: (1) What are the psychometric properties of a short version of the German (CCSS-S)? (2) Do the inter-rater reliabilities for the CCSS-S differ significantly between the expertise levels of raters (i.e., psychology students, advanced psychotherapy trainees, licensed psychotherapists)?

We assumed that the one-factor structure of the original CCSS, the internal consistency, and its nomological network associations could be replicated for the CCSS-S (23). In addition, we examined the influence of rater expertise on the ICCs. Based on previous results, one would assume that expert raters achieve higher ICCs. However, previous studies were based on comprehensive rating scales (CTS) (24), ACCS (25), while the CCSS was developed to be useful without intensive training. Thus, one could also argue that there should be equivalent results between different levels of rater expertise. For this reason, we conducted an exploratory comparison of the ICCs between psychology students, advanced psychotherapy trainees, and licensed psychotherapists.

## Materials and methods

We preregistered the methods and statistical analyses on the Open Science Framework^1^ and indicated when we deviated from the procedure described therein.

### Validation procedure for the clinical communication skills scale - short version

We analyzed four data sets that have recently been collected as part of three different studies (7, 23, 29). The data sets have not been analyzed before for the purpose of validating the CCSS-S. The validation was conducted in three parts (see text footnote 1 for a detailed description). (1) *Item Selection*: We analyzed the original data for the CCSS (data set 1, see below) to select the best items for a short version. (2) *Validation of the CCSS-S*: We analyzed three additional data sets (data sets 2–4, see below) to determine the validities (i.e., construct and convergent) and reliabilities (i.e., internal consistencies, intraclass correlations) for rater-based data. (3) *Exploratory analyses*: We compared the intra-class-correlations (ICCs) across different rater perspectives (psychology students, advanced psychotherapy trainees, licensed psychotherapists).

### Participants in the original studies

Data set 1 originates from a cross-sectional online study of which the main objective was to validate the CCSS (23). We selected the items for the CCSS-S based on an analysis of a subsample (*N* = 154) which evaluated the competences of a therapist in a video of a simulated therapy session (8 min). Data sets 2–4 were used for the validation of the CCSS-S. The data originate from two randomized controlled trials comparing different training methods for psychotherapists (7, 29). In each study, two trained raters watched videos of simulated therapy sessions (20 min) and evaluated the competences of *N* = 69 psychology students, in the roles of therapists across several measurement points. The raters were female and had different degrees of psychotherapy expertise: two licensed psychotherapists (data set 2), two psychology students (Master’s degree, data set 3), and two advanced psychotherapy trainees (data set 4). For the detailed design and sample descriptions, refer to the original studies (7, 23, 29).

### Ethics approval and consent to participate

Ethical approval for the original studies was obtained from the University of Potsdam Ethics Committee with the reference numbers 9/2018 (7), 01/2019 (23), and 60/2021 (29).

### Measurements

For the nomological network analysis, we examined (a) therapist competence with the German version of the Cognitive Therapy Scale (CTS) (20, 30), (b) therapist empathy with the German Empathy Scale (ES) (31), and (c) therapeutic alliance with the German Helping Alliance Questionnaire (32). All measurements are observer-based rating (ES and HAQ were originally developed as self-report instruments (33, 34), but we used the observer-based versions (for details on the measures, see Supplementary material 1).

### Statistical analyses

#### Item selection

The process of item selection is described in more detail in the pre-registration (see text footnote 1). A group of five experts (i.e., the study authors: three licensed psychotherapists, two psychologists with advanced psychotherapy training) selected and discussed those 20 items of the CCSS that best represented “clinical communication skills” (35). Statistical properties were also considered to ensure a balance between good representation of the construct, item difficulties (20–80%), and high item-total correlations (0.40–0.70) (36). Factor loadings ranged between 0.42 (Item 14) and 0.73 (Item 13), item difficulties ranged between 70 (Item 25) and 80% (Item 15), and item-total correlations ranged between 0.39 (Item 14) and 0.68 (Item 13). Finally, we selected 14 items for the CCSS-S (see Supplementary material 2). The CCSS-S needs approximately 2 min to complete.

#### Validation of the clinical communication skills scale - short version

The validation included a confirmatory factor analysis, a nomological network analysis, and a determination of the internal consistency and intraclass correlations (ICCs). Except for the ICCs, the rater scores were averaged across both raters per data set. All outcome variables were analyzed using mean scores. Analyses were conducted with R (37); Version 2021.09.1 + 372), including the packages lavaan (38) and psych (39).

##### Structural validity

###### Confirmatory factor analysis

In accordance with previous data on the CCSS (23), we specified a one-factor model using robust maximum likelihood estimation, and evaluated the model fit following the standard recommendations for the CFI, RMSEA, and SRMR fit indices (40): CFI ≥ 0.95, RMSEA ≤ 0.05– ≤ 0.06, SRMR ≤ 0.05– ≤ 0.08.

###### Nomological network analysis

We calculated bi-variate correlations for both the CCSS and the CCSS-S with the corresponding convergent measures. In the area of therapist competence, it is not easy to identify clear convergent and discriminant measures, because professional communication, empathy, and working alliance capture unique aspects but are still considered part of therapist competence (41). As already shown by other studies (23, 42), the intercorrelation between these variables is relatively high. Also, the performance of specific CBT techniques, as captured by the more global competency scales CTS or ACSS, cannot be separated from *the way* they are delivered (e.g., in what way and how emphatically they are communicated). Therefore, although we consider ES and HAQ to be conceptually discriminant measures for the CCSS-S in this study, we still expect moderate to high correlations. This is also suggested by the results of other studies (43, 44). To compare the nomological networks between the CCSS and the CCSS-S, we determined vector correlations based on the quantifying construct validity procedure (45), which “quantifies the match between a set of validity correlations and a set of hypotheses regarding convergent and discriminant validity” (p. 2). We interpreted the following two indicators in order to examine the degree of correspondence between the correlations of the CCSS and CCSS-S. Higher values (i.e., > 0.79) of the indicator *r*_*alerting*–*CV*_ indicate that the “degree to which the strongest (vs. weakest) predicted correlations are, in fact, the strongest (vs. weakest) actual correlations” (p. 6). In addition, higher values (i.e., > 0.71) of the indicator *r*_*contrast*–*CV*_ demonstrate “the degree to which the actual correlations are well differentiated (i.e., differ from each other) and are ordered (from high to low) in a way that parallels the predicted correlations.” (p. 7).

##### Reliability indices

We calculated the internal consistencies for the CCSS and CCSS-S using Cronbach’s alpha. In addition, we determined ICCs_(2,2)_ (46) for each group of rater pairs (i.e., psychology students, advanced psychotherapy trainees, licensed psychotherapists). We interpreted values less than 0.5 as “poor,” between 0.5 and 0.75 as “moderate,” between 0.75 and 0.9 as “good,” and greater than 0.90 as “excellent” (47).

#### Exploratory analyses: Rater-perspective comparison of intra-class-correlations

We compared the stability of the ICCs for the CCSS-S across different expertise levels of the raters (i.e., psychology students, advanced psychotherapy trainees, licensed psychotherapists). We concluded that ICCs were largely comparable across the various levels of expertise if the 95% CIs overlapped (48, 49).

### Sample size and power

The power calculations are described in detail in the pre-registration. We combined the data from the first two measurement points of data sets 2–4, leading to a sufficiently powered sample size of *N* = 690 competence ratings (see Supplementary material 3 for an overview of the sample sizes per data set).

### Deviations from the preregistration

We differed from the original pre-registration in the following ways: (1) Before conducting the analyses, we decided to refrain from using the Authenticity of Patient Demonstrations (50) as a discriminant measurement, because it is not related to therapist behavior but to the performance of simulated patients. (2) We analyzed *N* = 690 instead of *N* = 414 videos, as indicated in the pre-registration, because therapists in data sets 2 and 3 produced two videos (instead of one) per measurement point, due to there being two different tasks in the corresponding study (7). (3) We used Finn’s *r* as an additional inter-rater reliability coefficient, because, during the test of pre-requisites for using ICCs, we discovered that the data were skewed. In such cases, Finn’s *r* for ordinal data is recommended, because it is not influenced by low variances (51, 52); interpretation according to Pearson’s correlation) (4). Due to an unexpectedly poor model fit, we decided to calculate an exploratory structural equation modeling (ESEM) in addition to a confirmatory factor analysis (CFA).

## Results

The descriptive statistics and correlations for the CCSS and CCSS-S can be found in Table 1.

**TABLE 1:** Means, standard deviations, and correlations (*N* = 690).

Variable	*M*	*SD*	1	2	3	4
1. CCSS	2.93	0.44	−			
2. CCSS-S	2.88	0.47	0.97 (0.97, 0.98)	−		
3. CTS	2.55	0.87	0.82 (0.80, 0.85)	0.86 (0.84, 0.88)	−	
4. HAQ	3.20	0.71	0.89 (0.87, 0.90)	0.88 (0.86, 0.90)	0.82 (0.80, 0.85)	−
5. ES^a^	3.35	0.41	0.82 (0.79, 0.84)	0.80 (0.77, 0.83)	0.65 (0.60, 0.70)	0.82 (0.79, 0.85)

CCSS, Clinical Communication Skills Scale (original version); CCSS-S, Clinical Communication Skills Scale Short Version; CTS, Cognitive Therapy Scale; HAQ, Helping Alliance Questionnaire; ES, Empathy Scale; Values in brackets indicate the 95% confidence interval for each correlation. All values are significant at *p* < 0.001. ^*a*^*n* = 586 due to missing values.

### Factor structure

The one-factor CFA for the CCSS-S resulted in a borderline model fit, CFI = 0.90, RMSEA = 0.11, and SRMR = 0.05. Therefore, we decided to perform an additional exploratory analysis to better understand the factorial structure. We used exploratory structural equation modeling (ESEM) (53), which is intended to result in a more realistic representation of the data, because cross-loadings between items are allowed, just as in exploratory factor analysis, but not in CFA (54). To avoid bias that results from multiple analyses of the same data, we used a partly new data set for this analysis, namely the full data sets 2 and 3 including all three measurement points (*N* = 718 ratings; see Supplementary material 3).

Based on the procedure described by Silvestrin (55), ESEM starts with an exploratory factor analysis with oblique rotation, followed by an CFA-like model that implements cross-loadings, fixed factor variances, EFA loadings as starting points, and one anchor per factor (high loadings on one factor and low loadings on the others). The EFA yielded a two-factor solution (explained variance: Factor 1 = 22.5%, Factor 2 = 21.7%). The ESEM confirmed this structure, as indicated by an excellent model fit: CFI = 0.95, RMSEA = 0.07, and SRMR = 0.04. Factor 1 included 8 items (Supplementary material 4) with unstandardized loadings ranging from 0.39 (Item 1) to 0.84 (Item 3). This factor could be best described as *Collaboration Skills.* Factor 2 contained 6 items (Supplementary material 4) with loadings ranging from 0.46 (Item 4) to 0.96 (Item 5). This factor could be best described as *Structuring and Exploration Skills.* It is worth noting that there were relatively high cross-loadings, given that only five items loaded on their designated factor above 0.60. For example, Item 11 (“Works through content together with the patient”) in particular, had almost equal loadings on both factors (0.44 and 0.52). The two factors were significantly correlated (*r* = 0.67, *p* < 0.001). Because of the exploratory nature of this analysis, we did not use the two factors in the proceeding analyses.

### Nomological network analysis

Overall, there were high correlations between the CCSS-S and the other measurements (*r* = 0.80–0.88; Table 1). The vector correlations were *r*_alerting–*CV*_ = 0.80, indicating high similarity between the correlations of the original CCSS and the CCSS-S. The second vector index was very low, r_*contrast*–*CV*_ = 0.22, 95% CI [0.15, 0.30], *p* < 0.001. However, this is probably due to the restricted variance across the correlations, as we did not include discriminant measurements with low or only moderate correlations.

### Reliability and rater-perspective comparison

The reliability indices are displayed in Table 2. The internal consistencies of the CCSS-S were high (> 0.90). The ICCs ranged between moderate (data set 3, students), and good (data sets 2, experts, and 4, advanced trainees). In addition to the ICCs, we calculated Finn’s *r* for each item of the CCSS, because as Supplementary material 5 shows, the variance of CCSS scores was restricted and the data did not follow a normal distribution (except data set 4). For the CCSS-S items, Finn’s *r* ranged from 0.50 to 0.94, indicating good inter-rater reliability (Supplementary material 6). Although the student raters achieved the lowest ICC scores, the confidence intervals for the ICCs overlapped with experts and advanced trainees.

**TABLE 2:** Inter-rater reliabilities (ICCs) across the different rater perspectives.

	CCSS			CCSS-S		
Rater	ICC_(2, 2)_	95% CI	α	ICC_(2, 2)_	95% CI	α
Students^a^	0.70	(0.58, 0.78)	0.95	0.65	(0.51, 0.74)	0.92
Advanced Trainees^b^	0.80	(0.65, 0.88)	0.95	0.77	(0.65, 0.85)	0.91
Experts^a^	0.78	(0.71, 0.83)	0.97	0.75	(0.69, 0.81)	0.95

^*a*^*N* = 276, ^*b*^*N* = 138.

## Discussion

The purpose of the study was to validate the short version of the CCSS (23), the CCSS-S, which is an observer-based rating scale for the assessment of basic therapist skills, with a focus on communication. We pursued a transparent and structured approach to selecting appropriate items for the CCSS-S, following recommendations for constructing short scales (35). By analyzing a sufficiently large sample size (i.e., *N* = 690 video-based ratings of simulated therapy sessions), the results show that the CCSS-S is a feasible short scale that demonstrates comparable reliabilities and validity with the original scale.

The convergent correlations with other competence measurements (i.e., CTS, HAQ, ES) were high (*r*s > 0.86–0.89). The values demonstrate that each measurement can be subsumed under the construct of “therapist competence,” but still assesses certain unique aspects. While the HAQ focuses on the trusting relationship between patient and therapist, the ES focuses even more on the expression of empathy by the therapist. The CCSS-S also partially captures variables of empathy and therapeutic collaboration, for example with items such as “reacts with empathy to the feelings of the patient” and “works through the content together with the patient.” Similarly, the CTS includes items for assessing the alliance and empathy (i.e., interpersonal effectiveness), and also communication skills (i.e., guided discovery, clarity of communication, use of summaries). The high correlations among those measurements are in line with a recent meta-analysis of 53 studies, which found that the therapeutic alliance was significantly associated with therapist empathy (*r* = 0.50) (41). Furthermore, a longitudinal study demonstrated that the use of common factor skills, such as active listening, are associated with higher ratings of the alliance, and vice versa (56). Nevertheless, the description of the nomological network of “therapist competence” deserves further investigation, especially with regard to the discriminant variables. It has not yet been conclusively clarified whether different measurements with different competence foci are necessary, or whether it is simply very likely that competent therapists generally achieve high scores on different competence aspects [e.g., (16)].

The internal consistency of the CCSS-S (α = 0.91–95) was excellent and the inter-rater reliabilities in this study ranged between moderate to good [i.e., ICC_(2,2)_ = 0.65–0.80 at mean level, Finn’s *r*: 0.50–0.94 at item level]. In general, inter-rater reliabilities are lower when the variance of ratings is restricted (51), which was the case in the sample of this study. One explanation for this could be the standardized setting, in which the tasks for all participants were the same (e.g., in the student and expert rater data sets), the participants were mostly therapy beginners and thus had a similar skills level. In addition, raters were encouraged to consider the background knowledge of the participants. Future studies should examine the inter-rater reliabilities of the CCSS-S in real clinical situations, where a higher variance in skills can be expected.

One aim of the development of the instrument was not to afford a comprehensive rater training, because the items are behavior-based (23). Although the CCSS-S is in fact easy to administer, it might not be completely independent of the rater’s expertise. The ICCs between the different raters were comparable (as judged by their overlapping confidence intervals), however, the absolute ICCs were somewhat lower for the student raters. Future studies need to investigate this difference further. At the moment, it seems advisable to apply raters with at least some clinical experience to achieve good inter-rater reliabilities. However, if personal resources are limited, student raters are also a feasible option. In this case, close monitoring and additional training can improve the inter-rater reliabilities (57). This includes a shared understanding of the items and how to interpret behavioral indicators. All raters should be taught that competent communication involves a structured conversation in which one remains non-judgmental, speaks clearly and understandably, and works with the patient rather than giving him or her instructions.

Although the exploratory factor analysis of the original scale suggested a simple one factor model (23), the model fit of the CFA for the CCSS-S in this study was rather weak. It is quite likely that the restricted variance in the scores might have contributed to rather poor fit indices of the CFA. For this reason, we conducted an additional ESEM, resulting in a two-factor solution that represented the data very well. The two factors were labeled *Collaboration Skills* and *Structuring/Exploration Skills*. Such subscales are generally in line with other research conceptualizing CBT as an interplay between techniques and relationship skills [e.g., (58)]. Also, in analyzing the structure of the CTS, several authors suggested distinguishing similar factors, among others, that refer to structuring skills (e.g., agenda setting) and relationship skills [e.g., communication skills; (16, 59)]. However, the cross-loadings in the ESEM model were still relatively high for many items compared to the factor loadings. On the one hand, this might call into question the differentiation between the factors obtained. On the other hand, those cross loadings might simply display the conceptually logical interplay between most therapist skills; and ESEM models might thus be particularly useful to apply in the field of therapist competence. For example, therapists can’t structure the session without working collaboratively with patients. Therefore, only few items are unique indicators of the factors (i.e., Items 3, 10, and 14, giving time to speak, empathy, and clarifying as indicators of Collaboration; Items 5 and 6, summarizing and logic running through as indicators of *Structuring and Exploration*), whereas most skills have cross loading. Overall, the analysis of a more diverse therapist sample is desirable, before drawing final conclusions about the most appropriate factor structure of clinical communication skills as assessed with the CCSS-S.

### Limitations

The most important limitation is dependency within the data sets. To achieve the necessary power, we combined data from different studies and measurement points, some of which were based on assessment of the same participants (e.g., licensed psychotherapists in data set 2 and psychology students in data set 3 assessed the same participants). Although this procedure might bias the results, we are confident that the general correlational patterns will not be affected, because each video that is rated presents a new therapeutic situation. Thus, the factor structure of the CCSS-S and its correlations with other rating scales should not change. However, future research is needed to confirm the results obtained from this study with a larger data set of independent ratings. Another limitation is the lack of a suitable discriminant measurement to fully establish the validity of the CCSS-S. Future studies might include variables such as treatment adherence or behavior-based ratings of personality traits, such as extraversion (23). Fourth, the current validation relates to competence assessment in standardized role-play scenarios. Even though the authors of the original studies achieved a high level of authenticity in patient presentations, as measured *via* the rating scale for authentic patient demonstration (50), future studies should expand the validation and application of the CCSS-S to real therapy situations. Finally, measurements for alliance skills and empathy were based on scales that were originally used as self-reports, but observer-based versions are also available (32). Future studies should examine the convergent validities of the CCSS-S when it is also used as a self-report instrument, for example, when trainees are asked to self-assess their skills.

### Implications for training and research

The CCSS-S can be used for education and training purposes, and also in competence research. In the context of training, role plays are particularly suitable for assessing the communication skills of beginners using the CCSS-S. Our results suggest that advanced raters achieve higher levels of inter-rater reliabilities. However, given time and financial resources, it also seems appropriate to use student or peer raters to gain an impression of trainees’ skills. Nevertheless, all participants should familiarize themselves in advance, with the items of the scale. The CCSS-S might also be used as a self-assessment and reflection tool (29, 60). This way, the progress of trainees, their strengths and weaknesses in professional communication, could be monitored efficiently, for example, during the supervision process (3). There is also a growing interest in developing practical exams in which trainees demonstrate their skills in standardized role-plays, for example, the objective structured clinical examination (OSCE) is quite common in medical education (61). Although the CCSS-S has not yet been tested for creating summative assessments, we are confident that the scale can also be used for this purpose.

In addition to its application in education, we believe that the CCSS-S is also a useful tool for research, for example, as an efficient tool in longitudinal studies on therapist development. However, since reliability is particularly crucial for research purposes, we recommend the use of advanced raters over student raters. The training of these raters should follow the common recommendations for achieving high inter-rater reliabilities (57). Furthermore, the question of optimal training time is worth studying. The CCSS-S would prove particularly valuable if the time required not only to complete the scale but also to train raters could be reduced. This is also important for rater selection. For example, it might be reasonable to select clinically less experienced trainers who require more intensive training but are more affordable. In contrast, for some research questions, it might be important to use clinical experts as raters, who are more expensive but require less training effort to apply the CCSS-S.

Another important task for the field of competence research is the examination of associations between competence and patient outcomes. In their systematic review, Ottman et al. (11) noted that a positive relationship between competency and client outcomes was more prominent when common therapy factors (e.g., empathy) were used, in comparison to the assessment of specific CBT skills. Thus, future studies might also examine the associations between CCSS-S scores and client outcomes. In general, the next step for the research of competence assessment will be the application of the CCSS-S in real world conditions, that is, in real therapy situations (13). In addition, although the CCSS-S was developed as an instrument for general counseling and CBT skills (23), suitability for non-CBT contexts (e.g., psychodynamic therapies, acceptance, and commitment therapy) needs to be explored. Finally, the CCSS-S might also help in gaining a deeper understanding of deviations between self-reports and observer-based judgments (62).

## Conclusion

All in all, the CCSS-S is particularly useful for psychotherapy training using standardized role-plays, or general situations in which (a) general skills with a focus on communication skills are of interest, independent of specific CBT techniques, (b) when time resources are limited, and (c) when segments of therapy sessions should be evaluated. To ensure good inter-rater reliabilities for research contexts, we recommend employing raters with at least some clinical experience. For other contexts, such as peer evaluations, student raters achieve sufficient inter-rater reliabilities.

## Data availability statement

The original contributions presented in the study are included in the article, further inquiries can be directed to the corresponding author/s. The scripts of analysis are available at osf.io/xbeqa.

## Ethics statement

The studies involving human participants were reviewed and approved by the University of Potsdam Ethics Committee. The patients/participants provided their written informed consent to participate in this study.

## Author contributions

UM, FK, and FW conceived the study. UM wrote the study protocol and was responsible for the data analysis and for drafting the manuscript. All authors initiated and implemented the design, read, and agreed to the final manuscript.

## Footnotes

1
osf.io/xbeqa/?view_only=472979200d964de081ce45b141fd04f0
