# Supplementary material for: The concise measurement of clinical communication skills: Validation of a short scale
Source: Front Psychiatry. 2022 Oct 12;13:977324. doi: 10.3389/fpsyt.2022.977324 (PMC9596765; doi:10.3389/fpsyt.2022.977324)
Supplement: Supplementary file 1 [file Table_1.pdf]

Supplementary Material

1 Supplementary Figures and Tables

Supplementary material 1

Descriptions of the Measurements

Cognitive Therapy Scale. The German version of the Cognitive Therapy Scale (CTS; Weck et al., 2010; Young & Beck, 1980) assesses therapist competence. It consists of 14 items using a 7-point rating scale format: 0 = poor, 1 = barely adequate, 2 = mediocre, 3 = satisfactory, 4 = good, 5 = very good, and 6 = excellent. Depending on the data set, some items were excluded, as they could not be rated, given the specific task in the corresponding study (i.e., Data sets 2 and 3: Items 1, 7, 12; Data set 4: Item 12: Selection of appropriate strategies and Item 13: Appropriate implementation of techniques). The internal consistency for the German CTS was good in one study ($\alpha = 0.86$; Weck et al., 2010) and a previous meta-analyses reported fair to excellent values across several studies, ICCs = 0.42 – 0.97 (Kühne, Meister, et al., 2019).

Helping Alliance Questionnaire. The Helping Alliance Questionnaire (HAQ; Luborsky, 1984; Richtberg et al., 2016) measures the collaborative and affective bond between therapists and patients. It contains 11 items with a 6-point rating scale ranging from 1 = strongly disagree to 6 = strongly agree. Example items are "I believe the patient is working together with the therapist in a joint effort". The inter-rater reliability of the mean HAQ score between two raters was satisfactory in a previous study, $ICC_{(2,2)} = 0.73$; $p < 0.001$ (Richtberg et al., 2016).

Empathy Scale. For measuring therapist empathy, we used the German Version of the Empathy Scale (ES; Partschefeld et al., 2013; Persons & Burns, 1985). It contains 10 items on a 4-point rating scale ranging from 1 = strongly disagree to 4 = strongly agree. Examples of items are "The things my therapist says and does make me feel I can trust him.“ or "He understands my words, but not the way I feel.“ (item inverse coded). Partschefeld et al. (2013) report good internal consistencies between $\alpha = .84$ and $\alpha = .89$, and ICCs between .82 and .85.

Supplementary material 2

The Items of the CCSS-S

Clinical Communication Skills Scale – Short Version (CCSS-S)

These questions are aimed at capturing fundamental abilities in holding and formulating therapeutic conversations.

Please evaluate how appropriately the therapist applies the skills listed below. Note that the response scale ranges from the poorest score on the left to the best score on the right.

The therapist …	*Not at all appropriately*	*Not particularly appropriately*	*Generally appropriately*	*Entirely appropriately*
1. Makes use of appropriate facial expressions and gestures. (4)	◯	◯	◯	◯
2. Does not judge the patient. (11)	◯	◯	◯	◯
3. Gives the patient time to talk and to ask questions. (13)	◯	◯	◯	◯
4. Discusses content and procedure for the current session. (14)	◯	◯	◯	◯
5.Summarises interim results (15)	◯	◯	◯	◯
6. Ensures that the discussion has a logic running through it. (17)	◯	◯	◯	◯
7. Uses open questions to motivate the patient to talk. (19)	◯	◯	◯	◯
8. Uses closed questions to focus the discussion. (20)	◯	◯	◯	◯
9. Uses easily understandable language (21)	◯	◯	◯	◯
10. Reacts with empathy to the feelings of the patient. (25)	◯	◯	◯	◯
11. Works through content together with the patient. (26)	◯	◯	◯	◯
12. Explores thoughts, feelings and behaviour of the patient. (27)	◯	◯	◯	◯
13. Ensures that the patient understands by asking questions. (31)	◯	◯	◯	◯
14. Clarifies imprecise patient statements. (32)	◯	◯	◯	◯

Note. The numbers in brackets mark the item number of the original scale (Kühne et al., 2021)

Supplementary material 3

Overview of the Analyzed Data Sets

Study	People who were evaluated	Rater	Video Material	MP	No. of Videos for Analyses
CCSS-Original Study (Kühne, Heinze, et al., 2021)	1 therapist in demonstration video	**Data set 1**: n = 154 clickworker and general population	1 Therapy session with SP (8-9min)	1	**Item Selection: 154**
Skills Training Study (Kühne et al., 2020)	69 psychology students	**Data set 2**: n = 2 licensed psychotherapists<br>**Data set 3**: n = 2 students (M.Sc. psychology)	2 Therapy sessions with SP per MP (each 20min)	3	**Main analyses**: 69 participants x 2 sessions x 2 MP = 276 (**552** for Data sets 2 and 3 together)<br>**ESEM**[a]: 276 + 42 additional participants at MP 3 = 359 (one participant produced only 1 video: **718** for Data sets 2 and 3 together)
Live Supervision Study (Maaß et al., 2021)	69 psychology students	**Data set 4**: n = 2 advanced psychotherapy trainees	1 Therapy session with SP per MP (20min)	2	69 participants x 1 session x 2 MP = **138**

Notes. MP = Measurement points (The number of MP from data sets 2 and 3 varied according to the analysis in this study). CCSS = Clinical Communication Skills Scale. CCSS-S = Clinical Communication Skills Scale – Short Version. SP = standardized patient.

[a] At time of analysis, not all 69 participants had already completed MP 3; thus the sample size was lower than at MP 1 and 2.

Supplementary material 4

Factor Loadings of the ESEM-Two Factor Model for the CCSS-S

	Collaboration	Structuring and Exploration
CCSS3	**0.84**	-0.13
CCSS14	**0.63**	0.25
CCSS10	**0.61**	0.25
CCSS2	**0.55**	0.17
CCSS8	**0.54**	0.36
CCSS7	**0.50**	0.42
CCSS9	**0.41**	0.36
CCSS1	**0.39**	0.16
CCSS4	0.10	**0.46**
CCSS11	0.44	**0.52**
CCSS12	0.39	**0.54**
CCSS13	0.27	**0.59**
CCSS6	0.26	**0.61**
CCSS5	-0.12	**0.96**

Supplementary material 5

Descriptive Statistics and Test for Normality for the CCSS and CCSS-S per Rater

	CCSS										CCSS-S									
	Rater 1					Rater 2					Rater 1					Rater 2				
	M	*SD*	*Range*	*Mdn*	*p*	*M*	*SD*	*Range*	*Mdn*	*p*	*M*	*SD*	*Range*	*Mdn*	*p*	*M*	*SD*	*Range*	*Mdn*	*p*
Students	3.03	0.33	2.26-3.74	3.03	0.005	2.87	0.49	1.61-3.83	2.91	0.004	2.97	0.36	2.21-3.86	2.93	0.009	2.76	0.58	1.43-3.93	2.79	<.001
Adv. Trainees	**3.07**	**0.34**	**2.14-3.88**	**3.06**	**0.406**	**2.93**	**0.30**	**2.20-3.67**	**2.89**	**0.517**	2.92	0.38	1.93-3.86	2.86	0.045	**2.79**	**0.33**	**1.93-3.64**	**2.79**	**0.239**
Experts	2.81	0.56	1.44-3.93	2.86	<.001	2.98	0.65	1.00-4.00	3.03	<.001	2.80	0.59	1.21-3.93	2.86	<.001	2.98	0.67	1.00-4.00	3.03	<.001

Note. *p* = significance value of the Shapiro-Wilks test for normality. Adv. = Advanced. Bold = normal distribution. N's range between 138 (advanced trainees) and 276 (students, experts).

Supplementary material 6

Finn's r for the CCSS-S Items Separate for Rater Expertise

Items	Students	Advanced Trainees	Experts
CCSS1	0.80	0.84	0.73
CCSS2	0.80	0.87	0.70
CCSS3	0.69	0.84	0.71
CCSS4	0.63	0.74	0.60
CCSS5	0.71	0.79	0.66
CCSS6	0.66	0.82	0.70
CCSS7	0.75	0.78	0.65
CCSS8	0.86	0.86	0.68
CCSS9	0.74	0.90	0.81
CCSS10	0.77	0.85	0.69
CCSS11	0.72	0.78	0.68
CCSS12	0.74	0.90	0.73
CCSS13	0.71	0.80	0.55
CCSS14	0.68	0.94	0.50
